# Leptin Does Not Influence TSH Levels in Obese Short Children

**DOI:** 10.3389/fendo.2022.838881

**Published:** 2022-03-24

**Authors:** Katarzyna Adamczewska, Zbigniew Adamczewski, Andrzej Lewiński, Renata Stawerska

**Affiliations:** ^1^ Primary Care Unit “Medyk” – Outpatient Clinic, Ozorków, Poland; ^2^ Department of Nuclear Medicine, Medical University of Lodz, Lodz, Poland; ^3^ Department of Endocrinology and Metabolic Diseases, Polish Mother’s Memorial Hospital-Research Institute, Lodz, Poland; ^4^ Department of Endocrinology and Metabolic Diseases, Medical University of Lodz, Lodz, Poland; ^5^ Department of Pediatric Endocrinology, Medical University of Lodz, Lodz, Poland

**Keywords:** obesity, children, thyroid stimulating hormone, leptin, idiopathic short stature

## Abstract

**Introduction:**

Growth hormone (GH) and thyroid hormones are important for children growing. In some obese children a slightly elevated TSH concentration is observed. This may be an adaptive mechanism: stimulation of pro-TRH biosynthesis in the hypothalamus in response to elevated leptin. The increased TSH may also reflect the necessity of maintaining the resting energy expenditure or may be a result of inappropriate, low FT4 concentration. Thus, we evaluated serum TSH and FT4 concentrations in idiopathic short stature (ISS) children (non GH-deficient) and examined the effect of children’s nutritional status and levels of selected adipocytokines on thyroid function, searching for the presence of various forms of subclinical hypothyroidism, which may be the cause of the slow growth rate.

**Methods:**

The study group included 115 children (50 girls and 65 boys) with ISS, aged (mean ± SD) 10.4 ± 3.34 years. In each child, lipids, TSH, FT4, IGF-1, maxGH during the stimulation tests, leptin, adiponectin and resistin concentrations were determined. Based on BMI SDS, 3 subgroups: slim (n=26), obese (n=21) and normal weight (n=68) were distinguished.

**Results:**

There was no correlation between leptin level and TSH, FT4 levels. The levels of leptin, total cholesterol and LDL-cholesterol in obese short children were significantly higher than in children from other subgroups. In turn, the levels of adiponectin, resistin, TSH and FT4 did not differ between subgroups. In 7% of children, an elevated TSH level was found (but less than 10 mIU/L), with a similar frequency across subgroups. The higher the leptin, the lower maxGH in clonidine stimulation test was recorded.

**Conclusions:**

It seems that in obese children with idiopathic short stature leptin does not increase TSH secretion. This may be related to a disruption of the effect of leptin on TSH production and could indicate wide ranging disturbances of hypothalamic signals, and consequently be the cause of inappropriate GH secretion.

## Introduction

Growth hormone (GH), insulin-like growth factor 1 (IGF-1) and thyroid hormones play a key role in the growth processes in children. They act directly, however free T4 (FT4) and free T3 (FT3) also exert a permissive impact on IGF-1 action (1).

A child’s short stature may be due to hormonal causes (growth hormone deficiency - GHD, hypothyroidism or hypercortisolemia), chronic diseases (e.g. gastrointestinal diseases such as celiac disease) or some genetic syndromes (e.g. Turner or Prader-Willi syndrome). However, in many children, despite numerous tests, it is impossible to establish the cause of an inadequate high velocity and short stature. In such cases, idiopathic short stature (ISS) is diagnosed; however, it can be assumed that many of these cases are undiagnosed abnormalities caused by other factors involved in the regulation of the growth process (2).

It is known that many environmental factors and disruptors as well neuropeptides affect the production and secretion of TSH (3, 4). In our previous study, we proved a positive correlation between ghrelin and TSH concentrations in children with ISS. We also demonstrated that the higher the TSH, the lower the nocturnal GH and IGF-1 levels were recorded (5). Now, we have decided to analyse the effect of some selected hormones secreted from the adipose tissue (adipocytokines) on the secretion of TSH and FT4 in ISS children.

Subclinical hypothyroidism is observed in about 2% children (6, 7) and its diagnosis and treatment are a matter of controversy (8, 9); there are some conditions in which TSH elevation is transient, with obesity being one of the well documented examples (10). Many studies report that in children with excess body weight, a slightly elevated TSH level is observed (4-10 mIU/l), although it is not a response to reduced FT4 levels, i.e. not a result of hypothyroidism (10, 11). It has been hypothesized that it may be an adaptive mechanism aimed at increasing energy expenditure and the levels of TSH and FT4 correlate positively with resting energy expenditure (12). Thus, the elevated TSH levels in these cases seem a consequence rather than a cause of obesity, and treatment with levothyroxine in obese children is not recommended (12, 13). However, the causes of increased TSH in obesity are not clear and do not apply to all obese children (14). On the other hand, it was shown that among obese teenagers, the higher the TSH concentration, the higher total cholesterol and blood pressure values, which all are symptoms of hypothyroidism (15).

Therefore, the question arises: is the lack of an increase in TSH concentration in some obese children (without thyroid disease) also a normal phenomenon or, on the contrary, can it be the result of a too weak reaction of the hypothalamus and pituitary gland in TRH production/release in response to the body’s needs? In the latter case, abnormal hypothalamic and pituitary responses could explain short stature as an effect of relative FT4 deficiency.

The most popular hypothesis explaining the increase in TSH concentration in obese people is that leptin influences the production of pro-TRH in the hypothalamus (16, 17). Serum leptin concentration is strongly positively correlated with body weight and triggers a lot of actions connected with energy expenditure: at the level of the hypothalamus, it also inhibits appetite and increases hepatic gluconeogenesis and muscle fatty acids oxidation in peripheral tissues (18–20). The results of studies concerning leptin concentrations in children with GHD are divergent (21–24). It is possible that in some of them, the relative leptin resistance is observed. On the other hand, it was shown that leptin levels did not differ between children with hypothyroidism and hyperthyroidism, while significant differences were observed for adiponectin and resistin (respectively, higher and lower concentrations in untreated Graves’ disease than in hypothyroidism) (25).

Thus, it seems that the abovementioned adipocytokines (leptin, adiponectin and resistin) produced by adipose tissue may be involved in the cross-talk between adipocytes and hypothalamus, with the aim of increasing the release of TRH and TSH, and - in consequence – the production and secretion of FT4.

The aim of the study was to evaluate TSH and FT4 in idiopathic short stature (ISS) children, and to examine the effect of children’s nutritional status and levels of selected adipocytokines on thyroid function, in the search for various forms of subclinical hypothyroidism, which may be the cause of the slow growth rate.

Thus, the primary endpoint was the answer to the question how many children with short stature and obesity had elevated TSH levels co-occuring with elevated leptin levels. And - in those who do not have elevated TSH - is it associated with symptoms of hypothyroidism other than short stature, e.g. elevated cholesterol concentration?

## Materials and Methods

The study included consecutive children admitted to the Department of Endocrinology and Metabolic Diseases of Polish Mother’s Memorial Hospital - Research Institute in Lodz over the period of one year for the diagnostics of their short stature, who met the following criteria:

1. height standard deviation score (HSDS) below -2.0 from the mean value for child’s age and sex (children’s height was measured using a stadiometer) (26);

2. excluding genetic reasons of the short stature (i.e. Turner syndrome, Prader-Willi syndrome) (assessed on the basis of a karyotype);

3. excluding treated hypothyroidism, chronic diseases or undiagnosed gastrointestinal tract complaints (assessed on the basis of a negative history of chronic diseases, as well as normal tests results of tissue transglutaminase antibodies class IgA).

Out of the initially analyzed group of 170 children: 10 did not meet the criterion of height below 3 centile, 7 did not meet the criterion of low height velocity, 4 had treated hypothyroidism, and 1 - celiac disease. Ultimately, 148 short children were qualified for further analyses.

The body mass was assessed in all patients, and that was followed by a calculation of the body mass index standard deviation score for chronological age (BMI SDS for CA). We used Polish references (26). Next, in all the children, considering the child’s current position on centile charts, the height age (HA) was calculated (as the age ascribed to the 50th percentile for a given child’s height) and BMI values reffered to HA and expressed as BMI SDS for HA (we adjusted the results to the height age of a child to avoid false results for short children). Based on this value, the analyzed group of short children was divided into three subgroups (according to WHO recomendatios): obese, normal and slim. The obesity and overweight group includes children aged 5–19 with BMI for HA above +1.0 SD (above 90 percentile) and children under 5 years of age with BMI for HA above +2.0 SD (above 97 percentile). Into the slim group, we qualified children with thinness: BMI SDS for HA <-2.0 SD (below the 3rd percentile), regardless of their chronological age.

The stage of puberty was assessed in each child, using the Tanner**’**s scale. Most of the analyzed children were prepubertal (83%).

In all of them, GH secretion was assessed during a 3-hour nocturnal profile and during two (2) stimulation tests: the first one after clonidine administered orally (with the dose of 0.15 mg/m^2^ of the body surface) and GH concentration measurements at time 0 and at the 30th, 60th, 90th and 120th minute of the test, and the second one – after intramuscular administration of glucagon (in the dose of 30 µg/kg of body weight, not exceeding 1 mg), with GH concentration measurements at time 0 and at the 90th, 120th, 150th and 180th minute. Based on the results of GHmax values in these tests, we diagnosed:

1. ISS – correct results in - at least - one of the stimulation tests (GHmax values ≥ 10 ng/ml) in 115 children (50 girls and 65 boys),

2. GHD – decreased GH secretion (GHmax values < 10 ng/ml) in 33 children.

In each child, the morning serum cortisol and ACTH levels were routinely assessed to rule out secondary adrenal insufficiency, while in obese children, also the cortisol profile (or a dexamethasone test) was performed to rule out hypercortisolemia. These disorders were not found in any of the children. We also routinely assessed the levels of anti thyroglobulin (a-Tg) and anti thyroid peroxidase (a-TPO) antibodies, they were normal in every child. In each child, the concentration of IGF-1, IGFBP-3, lipids, TSH, FT4, leptin, adiponectin and resistin was assessed in the fasting state on the first day of hospitalisation, just before the first stimulating test. Next, IGF-1 concentrations were calculated as IGF-1 SDS, according to the reference data (27). For the calculation of IGF-1/IGFBP-3 molar ratio, the following molecular masses were used: 7.5 kDa for IGF-1 and 42.0 kDa for IGFBP-3. For IGF-1/IGFBP-3 molar ratio, the cutoff point was established at the median values.

Growth hormone levels were measured using the immunometric method. The measurements were performed by Immulite, DPC assay kits, calibrated to the WHO IRP 98/574 standard set, of the following sensitivity level: 0.01 ng/ml, range: up to 40 ng/ml, the conversion index: ng/ml x 2.6 = mIU/l, the intra-assay CV: 5.3-6.5% and inter-assay CV: 5.5-6.2%.

Both IGF-1 and IGFBP-3 concentrations were assessed by Immulite, DPC assays; WHO NIBSC 1st IRR 87/518 standard was applied, with the analytical sensitivity of 20 ng/ml, calibration range up to 1600 ng/ml, the intra-assay CV: 3.1-4.3% and inter-assay CV: 5.8-8.4%. The assay for IGFBP-3 assessment was calibrated to WHO NIBSC Reagent 93/560 standard, with analytical sensitivity 0.02 μg/ml, the calibration range up to 426 μg/ml, the intra-assay CV: 3.5-5.6% and the total CV: 7.5 9.9%.

The leptin, resistin and adiponectin concentrations were measured using the Millipore Elisa kit (Linco Research, USA). The sensitivity level, intra-assay CV and inter-assay CV were: 0.5-100 ng/ml, 1.4-4.9% and 1.3-8.6% for leptin; from 0.16 ng/ml, 3.2-7.0% and 7.1-7.7% for resistin and from 0.78 ng/ml, 7.4% and 2.4-8.4% for adiponectin, respectively.

Concentrations of TSH and FT4 were measured by the electrochemiluminescent immunoassays (ECLIA) method with commercially available appropriate kits (Roche Diagnostic, Mannheim, Germany). Normal range values were as follows: for TSH: age-dependent ranges - 1–7 years-0.7–5.97 mIU/l; 7–12 years-0.6–4.84 mIU/l; 12–18 years-0.51–4.4 mIU/l with inter-assay coefficients of variation (CVs) 1.3–1.8% and for FT4: age-dependent ranges-1–6 years-0.96–1.77 ng/dl; 6–11 years-0.97–1.67 ng/dl; 11–18 years-0.98–1.63 ng/dl with CVs 2.0–2.4%.

The data were analyzed using Statistica 11.0 software (StatSoft, Inc., Tulsa, OK, USA). The continuous variables were expressed as mean ± standard deviation for normally distributed variables. Shapiro-Wilk**’**s test was used to test the distribution of the variables. Correlations were evaluated using the Pearson**’**s test. A one-way ANOVA was applied for statistical analysis with the subsequent use of a *post-hoc* test, in order to statistically assess differences between groups; Tukey**’**s test was selected because of the uneven amount of data in individual groups. p < 0.05 was accepted as significant value.

## Results

Among 115 children (50 girls and 65 boys), aged 3.66 to 16.52 yrs; the mean age **±** SD: 10.43 ± 3.34 years with ISS, we found: 26 slim children, 68 – with normal body weight and 21 - overweight or obese. The results of the auxological parameters and the laboratory tests results for individual subgroups (divided by BMI values) are presented in **Table 1**.

**Table 1:** The results of analysed parameters in individual subgroups (slim, normal and obese) of ISS children.

	Slim	Normal	Obese	Variance analysis
n = (girls/boys)	26 (14/12)	68 (25/43)	21 (11/10)	
Chronological age (CA) (years)	9.60 ± 3.12	10.56 ± 3.12	10.26 ± 3.31	ns
Height age (HA) (years)	7.18 ± 2.38	8.29 ± 2.62	7.59 ± 2.70	ns
HSDS	-2.71 ± 0.97	-2.53 ± 0.82	-2.53 ± 0.88	ns
BMI (kg/m^2^)	13.41 ± 0.80^a,b^	16.33 ± 1.44^a,c^	20.40 ± 2.94^b,c^	<0.0005
BMI SDS for CA	-1.28 ± 0.42^a,b^	-0.46 ± 0.48^a,c^	1.43 ± 0.95^b,c^	<0.0005
BMI SDS for HA	-1.27 ± 0.34^a,b^	-0.03 ± 0.41^a,c^	2.05 ± 1.01^b,c^	<0.0005
TSH (mIU/l)	2.71 ± 1.30	2.53 ± 1.14	2.69 ± 1.52	ns
FT4 (ng/ml)	1.36 ± 0.12	1.31 ± 0.20	1.3 ± 0.14	ns
TG (mg/dl)	74.05 ± 27.32	64.85 ± 25.07	74.29 ± 31.54	ns
CH (mg/dl)	150.00 ± 34.72	160.00 ± 28.39	175.56 ± 49.46	<0.05
LDL-CH (mg/dl)	83.58 ± 28.25^a^	85.62 ± 26.62^b^	108.21 ± 36.71^a,b^	<0.05
HDL-CH (mg/dl)	55.58 ± 19.26	60.72 ± 16.54	60.79 ± 11.91	ns
HDL/CH	0.36 ± 0.10	0.39 ± 0.09	0.34 ± 0.08	ns
Leptin (ng/ml)	2.44 ± 5.11^a^	4.59 ± 5.04^b^	11.86 ± 11.47^a,b^	<0.0005
Adiponectin (ng/ml)	18.43 ± 6.97	17.77 ± 8.30	19.80 ± 11.43	ns
Resistin (ng/ml)	9.91 ± 4.05	10.48 ± 4.01	9.13 ± 2.31	ns
Leptin/Adiponectin ratio	0.07 ± 0.4^a^	0.35 ± 0.4^b^	0.73 ± 0.43^a,b^	<0.0005
maxGH after clonidine (ng/ml)	19.50 ± 9.59^a^	17.43 ± 8.70^b^	11.75 ± 6.63^a,b^	<0.05
maxGH after glucagon (ng/ml)	10.81 ± 7.80	9.86 ± 7.03	12.61 ± 6.02	ns
maxGH during sleep (ng/ml)	16.47 ± 9.31	13.89 ± 8.95	10.86 ± 5.52	ns
IGF-1 (ng/ml)	120.26 ± 65.57^a,b^	192.30 ± 116.67^a^	216.79 ± 120.01^b^	<0.01
IGFBP-3 (μg/ml)	3.81 ± 1.09	4.47 ± 1.13	4.81 ± 1.84	ns
IGF-1/IGFBP-3 molar ratio	0.16 ± 0.06^a^	0.23 ± 0.12^a^	0.24 ± 0.11	<0.05
IGF-1 SDS	-1.22 ± 1.19	-1.10 ± 1.18	-0.53 ± 0.78	ns

^a,b,c^values in the same row with different superscripts are significantly differed (p<0.05); BMI – body mass index, SDS – standard deviation score, TG - triglycerides, CH – cholesterol, IGF-1 – insulin-like growth factor 1, IGFBP-3 – insulin-like growth factor binding proteins 3, maxGH – maximal GH concentration during stimulation tests or during sleep.

TSH levels were slightly elevated in 8 children: including 2 out of 26 slim children (7.7%), 2 out of 21 obese children (9.5%) and 4 out of 68 normal weight children (5.9%); FT4 level was in normal range in each of these cases.

As expected, the levels of leptin in obese children were significantly higher than in the other groups, but the levels of adiponectin and resistin did not differ between groups.

The degree of growth deficiency and the other (except leptin) test results did not differ between subgroups.

Mean FT4 concentration was the lowest in the subgroup of obese children, but the values did not reach statistical significance (**Table 1**).

In the whole group of ISS children, we observed a positive significant correlation between adiponectin and FT4 concentration (**Table 2**). There was no significant correlations of the body mass index (i.e. BMI SDS for CA or for HA), leptin, adiponectin or resistin concentration with TSH and FT4 concentrations. However, we noticed significant positive correlations between leptin/adiponectin ratio and: cholesterol, LDL-fraction of cholesterol and triglicerides (**Table 2**).

**Table 2:** The correlation of TSH, FT4 and lipids concentration with BMISDS and selected adipocytokines.

	BMI SDS for HA	Leptin	Adiponectin	Leptin/adiponectin ratio	Resistin
TSH	0.06	-0.09	+0.04	-0.13	+0.07
FT4	-0.02	-0.02	+ 0.2*	-0.18	+0.1
Cholesterol	+0.21	+0.2	+0.03	+0.29*	-0.19
LDL-Cholesterol	+0.24*	+0.14	-0.03	+0.26*	-0.12
HDL-Cholesterol	+0.14*	+0.07	+0.21	0.05	-0.15
Triglicerides	-0.04	+0.26*	-0.05	+0.28*	+0.11

^*^p < 0.05; BMI SDS for HA - body mass index standard deviation score for height age

It was observed that the higher the leptin the lower the GH secretion during the test with clonidine (r=-0.32, p < 0.05), as well as the negative correlation between leptin/adiponectin ratio and GH secretion during the test with clonidine (r=-0.39, p < 0.05) and during sleep (r=-0.2, p < 0.05).

## Discussion

In the group of children with ISS included in our study, no increase in TSH serum level was observed with increasing children**’**s BMI and leptin concentration. In the subgroup of obese short children, TSH levels were not higher than in other subgroups. Although the group we studied was small (which is a limitation of our work), it seems that our results are worth showing. Many studies on both children and adults, conducted on large groups of patients, have shown that TSH levels correlate positively with BMI and leptin (28, 29). However, an interesting aspect that distinguishes our study is that it concerned children with short stature. Higher leptin concentrations may partially explain the effect of obesity on thyroid function, perhaps through the effect of leptin on TSH secretion, as this increase has been shown to be correlated with leptin regardless of BMI (28, 29). Thus, it is surprising that we did not find such a relationship in our group. Although the thesis concerning the increase in TSH in obesity due to the increased production of pro-TRH through the stimulation of the hypothalamus by leptin is plausible, there are certainly other mechanisms that influence (modulate) this relationship. One of them may be the excessive concentration of ghrelin, which we wrote about in the previous work (10). It is also possible that inflammation drives the changes in TSH and thyroid hormone levels in obesity. Weight reduction is likely to be associated with a reduction in inflammation and may explain the observed correlation between weight loss and a reduction in TSH (12). We have also recently analysed the prevalence of elevated TSH in children with acute respiratory infection and found elevated TSH in 10% of the cases, which returned to normal in all children shortly after recovery (30).

The slightly elevated TSH levels are seen in obese individuals, but not in all of them (12, 31–33). In a study by Habib et al. (5), TSH and FT4 levels were assessed in 850 children aged 2 to 18 years, and it was found that elevated TSH levels are observed in 17.2% of overweight and 20.5% of obese children; in contrast to 9.9% of slim and only 3.8% of normal weight children. In turn, in the study by Wolters et al. (12), elevated TSH was observed in 39% of 477 obese children, however the authors set the cut-off point for the elevated TSH concentration at a lower level, i.e. 3.0 mIU/l.

Thus, the prevalence of hyperthyrotropinemia among obese patients differs in individual analyses.

Meanwhile, in 2020, Wang et al. (11) found that increased TSH levels are more often observed in girls with generalized obesity compared to those with central obesity. We did not analyse the type of obesity in our research. It should also be taken into account that among our patients there were no patients with extreme obesity. The highest value of BMI was +3.9 SD.

In 2019, Ruszała et al. (14) assessed the influence of the thyroid axis dysfunction on the occurrence of metabolic obesity complications. They analysed a group of 100 obese children, where 25 children had features of the metabolic syndrome and 75 did not. The authors found no case of overt thyroid disease within the whole analyzed group. There were no significant differences in mean TSH, FT4, and FT3 levels in patients with and without the metabolic syndrome. Moreover, an elevated TSH level was found in 8% of obese patients with the metabolic syndrome and 24% of obese patients without it. The authors concluded that an isolated increased TSH level is not common in obese adolescents and there is no correlation between TSH, FT3, FT4 levels and BMI SDS value. Moreover, isolated increased TSH levels were not associated with the occurrence of the metabolic syndrome in obese adolescents (14).

In the present study, we also analysed lipids profile. We found a significantly higher concentration of total cholesterol and LDL-cholesterol in the subgroup of children with obesity. However, we did not find any significant correlation between lipids and each of the analysed hormones (i.e. TSH or FT4). In particular, a positive correlation between proatherogenic lipids (cholesterol, LDL-cholesterol and triglycerides) concentrations and leptin/adiponectin ratio was found. It may suggest that the metabolic disorders which we observed were the result of too weak stimulation of pro-TRH and TSH by leptin (e.g, in certain disorders at the hypothalamic level) and, in consequence, the relative hypothyroidism. It may also be a possible explanation of an additional observation we made, namely a negative correlation between leptin concentration (or leptin/adiponectin ratio) and GH secretion during the stimulation test with clonidine. This phenomenon, observed in obese children, can be explained - among others - by the blocking effect of lipid disorders on GHRH and GH secretion (34, 35). Therefore, it is possible that some obese children experience a weaker action exerted by TRH and GHRH jointed on the level of the pituitary gland.

In turn, Bossowski et al. (25) explored other aspects of these issues. They analysed leptin, adiponectin and resistin in children with untreated Graves**’** disease and hypothyroidism in Hashimoto**’**s thyroiditis. The authors showed higher adiponectin and lower resistin levels in hyperthyroidism than in hypothyroidism. The analysis of leptin levels revealed no significant differences between children with subclinical hypothyroidism and untreated Graves**’** disease. Thus, their research also supports the idea that leptin and TSH levels are in a poor cause-and-effect relationship. However, they suggested that disturbances in thyroid hormones in thyroid diseases have a significant effect on the levels of adiponectin and resistin released by adipose tissue (25). We also observed the same relationship between FT4 and adiponectin concentrations in the analysed group of children. It is to be noted that a higher FT4 concentration should be beneficial for the decreased leptin/adiponectin ratio. However, in the analysed group of short children we did not find that relationship. Thus, the observed phenomenon of increased TSH in some obese children is probably multifactorial, and in children without thyroid disease could trigger protective effects, but it does not seem to apply to all obese children and especially to obese children with idiopathic short stature.

The lack of leptin influence on TSH concentration could indicate wide ranging disturbances of hypothalamic signals, and consequently be the cause of inappropriate GH secretion.

## Data Availability Statement

The original contributions presented in the study are included in the article/supplementary material. Further inquiries can be directed to the corresponding author.

## Ethics Statement

The studies involving human participants were reviewed and approved by the Bioethical Committee at the Polish Mother’s Memorial Hospital-Research Institute (PMMH-RI) in Lodz. Written informed consent to participate in this study was provided by the participants’ legal guardian/next of kin.

## Author Contributions

Conceptualization, KA and RS. Methodology, RS and KA. Resources, RS. Writing—original draft preparation, KA and ZA. Writing—review and editing, AL. Supervision, AL and RS. All authors have read and agreed to the published version of the manuscript.

## Funding

This research was funded by the National Science Centre Poland (NCN), grant number 01030 P05E and by statutory funds from the Medical University of Lodz, Lodz, Poland (503/1-107-03/503-11-001-19-00).

## Conflict of Interest

The authors declare that the research was conducted in the absence of any commercial or financial relationships that could be construed as a potential conflict of interest.

## Publisher’s Note

All claims expressed in this article are solely those of the authors and do not necessarily represent those of their affiliated organizations, or those of the publisher, the editors and the reviewers. Any product that may be evaluated in this article, or claim that may be made by its manufacturer, is not guaranteed or endorsed by the publisher.
